# AUtomated Risk Assessment for Stroke in Atrial Fibrillation (AURAS-AF) - an automated software system to promote anticoagulation and reduce stroke risk: study protocol for a cluster randomised controlled trial

**DOI:** 10.1186/1745-6215-14-385

**Published:** 2013-11-13

**Authors:** Tim A Holt, David A Fitzmaurice, Tom Marshall, Matthew Fay, Nadeem Qureshi, Andrew R H Dalton, F D Richard Hobbs, Daniel S Lasserson, Karen Kearley, Jenny Hislop, Jing Jin

**Affiliations:** 1 Department of Primary Care Health Sciences, University of Oxford, Radcliffe Observatory Quarter, Woodstock Road, Oxford, OX2 6GG, England; 2 Primary Care Clinical Sciences, School of Health and Population Sciences, College of Medical and Dental Sciences, University of Birmingham, Birmingham, B15 2TT, England; 3 Public Health, Epidemiology and Biostatistics, College of Medical and Dental Sciences, University of Birmingham, Birmingham, B15 2TT, England; 4 Westcliffe Medical Centre, Westcliffe Road, Shipley, BD18 3EE, England; 5 School of Community Health Sciences, University of Nottingham, University Park, Nottingham, NG7 2RD, England

**Keywords:** Stroke, Atrial fibrillation, Prevention, Reminder systems, Anticoagulants, Primary care, Electronic medical records

## Abstract

**Background:**

Patients with atrial fibrillation (AF) are at significantly increased risk of stroke. Oral anticoagulants (OACs) substantially reduce this risk, with gains seen across the spectrum of baseline risk. Despite the benefit to patients, OAC prescribing remains suboptimal in the United Kingdom (UK). We will investigate whether an automated software system, operating within primary care electronic medical records, can improve the management of AF by identifying patients eligible for OAC therapy and increasing uptake of this treatment.

**Methods/Design:**

We will conduct a cluster randomised controlled trial, involving general practices using the Egton Medical Information Systems (EMIS) Web clinical system. We will randomise practices to use an electronic software tool or to continue with usual care. The tool will a) produce (and continually refresh) a list of patients with AF who are eligible for OAC therapy - practices will invite these patients to discuss therapy at the start of the trial - and b) generate electronic screen reminders in the medical records of those eligible, appearing throughout the trial. The software will run for 6 months in 23 intervention practices. A total of 23 control practices will manage their AF register in line with the usual care offered. The primary outcome is change in proportion of eligible patients with AF who have been prescribed OAC therapy after six months. Secondary outcomes are incidence of stroke, transient ischaemic attack, other major thromboembolism, major haemorrhage and reports of inappropriate OAC prescribing in the data collection sample - those deemed eligible for OACs. We will conduct a process evaluation in parallel with the randomised trial. We will use qualitative methods to examine patient and practitioner views of the intervention and its impact on primary care practice, including its time implications.

**Discussion:**

AURAS-AF will investigate whether a simple intervention, using electronic primary care records, can improve OAC uptake in a high risk group for stroke. Given previous concerns about safety, especially surrounding inappropriate prescribing, we will also examine whether electronic reminders safely impact care in this clinical area.

**Trial registration:**

http://ISRCTN 55722437

## Background

Despite improving trends in incidence, stroke remains a major health burden in the United Kingdom (UK), and internationally. Cerebrovascular disease is still the second largest cause of mortality in England and Wales [1], and has a greater disabling impact than any other chronic disease [2]. Improved management of risk factors has been central to recent improvements in outcomes [3], but a notable exception is the management of stroke risk in patients with atrial fibrillation (AF).

AF is a major independent risk factor for stoke, conveying up to a five-fold increased risk [4]. AF-associated strokes are generally more severe, with greater case fatality and subsequent disability than those not associated with AF [5]. Anticoagulation is established as an effective method of reducing stroke risk (by up to 67%) in patients with AF [6,7]. Despite the evidence of benefit, however, there remains considerable underuse. Recent studies place UK prescribing at as low as 53 percent of those eligible (based on national clinical guidelines of the time) [8], with up to 34 percent not prescribed and without a recorded contraindication or refusal [9]. This underuse is also evident internationally [10,11]. A number of factors contribute to this, including clinician misconceptions over risk of haemorrhage, especially in older people; time implications of the management of the most commonly used anticoagulant, warfarin; and confusion over eligibility [12]. There are risks from anticoagulation, notably haemorrhage, and some patients at high risk of bleeding should be excluded from this treatment. The pros and cons of anticoagulation are, however, often misjudged by clinicians, and based on the balance between haemorrhage and thromboembolism risk, most should be considered for OACs [13].

Decision support tools have been highlighted as one option to promote evidence-based prescribing of anticoagulants in AF [14]. Patient-specific electronically generated reminders - those which draw on routine medical record data to generate reminders specific to a patient - produce moderate, but significant, improvements in clinical processes, including prescribing [15]. However, concerns remain over their use, including the appropriateness of discussing issues outside the patient’s agenda during a consultation (resulting in a loss of patient autonomy) [16]; their impact on consultation length and flow [17]; and clinicians developing a reliance on them [18]. In the case of OAC therapy in AF, there is an added risk that an important decision might be rushed in the opportunistic setting.

We aim to develop an intervention to promote oral anticoagulation in eligible patients with AF (defined by the National Institute for Health and Clinical Excellence (NICE) guidance TA249 [19]), and further to test its effectiveness and safety. We do not differentiate between types of OAC (for example the most commonly used warfarin, or newer OACs such as dabigatran). Our intervention builds on the approach of the existing GRASP-AF tool [9] (drawing on routinely collected primary care data to identify eligible individuals with AF for OAC), but in addition will automatically update itself by repeatedly interrogating the practice database, and will be linked to screen reminders. Our intervention follows the established principle of repeated, electronically embedded audit cycles in primary care, typified by the UK Quality and Outcomes Framework (QOF) that supports the care of the more common chronic diseases [20]. Critically, to our knowledge, no such tool has been previously assessed in a randomised trial in this area of care.

### Aim

The aim of this trial is to investigate the impact of an automated system to identify patients with AF who are eligible for oral anticoagulant therapy (defined by NICE guidance TA249 [19]) to reduce stroke risk and to identify barriers to commencing recommended care using the tool.

### Objectives

The objectives of this trial are as follows:

1. To examine whether an automated software system operating within electronic medical records to identify patients with AF who are eligible for oral anticoagulant therapy will increase uptake.

2. To investigate whether the electronic software system can produce short term reductions in thromboembolism in high risk patients with AF.

3. To examine the safety of the electronic software system in promoting anticoagulation in patients with AF, including its impact on inappropriate prescribing and haemorrhages.

4. To examine patient and practitioner views of electronic systems that incorporate automated reminders in primary care and their impact on the primary care consultation and practice.

5. To investigate barriers and enablers to the successful uptake of an electronic audit and reminder tool to promote anticoagulation in patients with AF by primary care teams.

6. To explore reasons why patients with AF are not prescribed anticoagulants and identify any barriers to recommended prescribing that persist after the use of an electronic audit and reminder system.

## Methods/Design

### Study design

**Figure 1:**
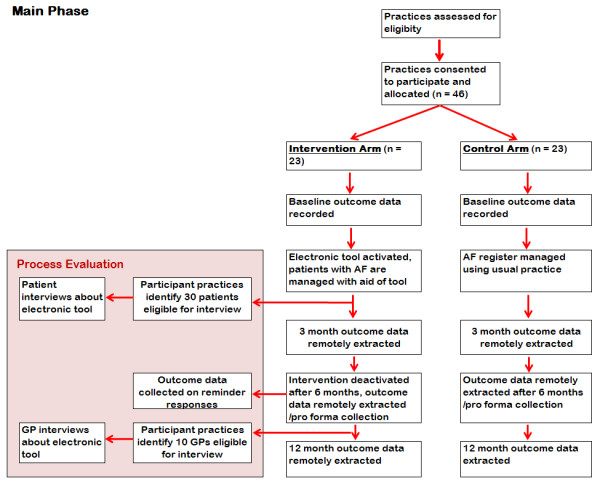
**Outline design of the AURAS-AF trial.** AF = Atrial Fibrillation; GP = General Practitioner.

We will use a cluster randomised controlled trial (Figure 1) to investigate whether a decision support tool in electronic primary care records safely increases anticoagulant prescribing in eligible patients with AF. The general practice is the unit of allocation and study participant because the intervention aims to improve prescribing at a practice level. The intervention phase will last for 6 months, with data collection immediately following, and after a further 6 months. Control practices will undergo pre-/post-assessment, but will not receive the active software tool. We shall conduct a 3-month pilot study before the main phase, and a process evaluation in parallel with it.

### Study setting and population

The study is set in English general practices, situated within the South East of England, Central England, and East Midland and South Yorkshire Primary Care Research Networks (PCRNs), which use the EMIS Web clinical system. We exclude practices that have carried out a major audit of anticoagulant prescribing (including the systematic invitation of patients) within the last 2 years; those previously involved in research into anticoagulation (including those involved in the pilot study) or those who have begun using the EMIS Web system within the last 3 months. EMIS are the largest among several providers of clinical software to UK general practice and their Web version is becoming widely adopted across the UK. Study practices will be recruited through a letter of invitation sent from the study team, through the local PCRN, with a practice guardian providing consent [21,22]. All practices using EMIS Web within the study region will be given equal opportunity to participate in the study.

### Intervention

The intervention is an electronic software tool installed in practice clinical systems. The tool identifies patients with AF who are eligible for anticoagulants based on recent guidelines (NICE TA249) [19], and are not currently prescribed therapy. We have designed the tool to operate in the EMIS Web clinical system. The tool will identify eligibility by interrogating data held in patient medical records. The NICE TA249 eligibility criteria are operationalised using the relevant clinical codes (see Additional file 1). Given that some criteria defining eligibility (see below) may be poorly coded, the tool highlights (through screen messages) patients with suspected relevant diagnoses (diabetes, heart failure, or hypertension), based on other aspects of their medical record (detailed decision rules available from the authors). These must be addressed by the clinician, with the diagnosis either confirmed or rejected, before proceeding. For instance, in current practice, most patients with known, symptomatic heart failure will be on the QOF Heart Failure register, a register that requires echocardiographic evidence of reduced left ventricular ejection fraction or a diagnosis of heart failure by a cardiologist. It is very unlikely that a patient will be on this register if they have neither NYHA class 2 symptoms (or greater) nor echocardiographic evidence of a low ejection fraction. However, in cases not on the register but where clinical codes suggest they should be, this will be questioned using automatically generated screen messages. Similar decision rules apply to possible uncoded diabetes and hypertension. In addition, the tool requests clarification over the nature (thromboembolic or haemorrhagic) of past recorded strokes, where non-specific electronic codes have been applied to the record. This is important as the distinction has a very important influence on eligibility for OAC therapy. This clarification is obtained by responses to screen prompts and these responses will be collected during the study.

The tool has two components:

1. A search module that maintains a list of patients diagnosed with AF, highlighting the group eligible for anticoagulation under current recommendations and who are currently not prescribed OAC therapy. According to NICE TA249 [19] patients are considered eligible if they meet one or more of the following criteria:

••previous stroke, transient ischaemic attack or systemic embolism

••left ventricular ejection fraction below 40%

••symptomatic heart failure of New York Heart Association (NYHA) class 2 or above

••age 75 years or older

••age 65 years or older with one of the following: diabetes mellitus, coronary artery disease or hypertension.

Once activated, the software will automatically refresh the list every 24 hours using data in the electronic medical records.

2. It will generate onscreen reminders, prompting clinicians (either general practitioners or practice nurses) about the need for anticoagulation in patients with AF. Screen reminder messages will appear whenever the electronic record of a patient eligible for, but not currently being prescribed anticoagulants, is opened by a clinician. These reminders must be actively responded to, with the clinician choosing from a number of options as indicated below. Options 1 and 2 will remove the screen message, but it will return next time the record is opened; options 3 and 4 will remove the screen message, which will not reappear as long as the corresponding ‘Read’ code is entered into the record or an OAC is prescribed respectively; option 5 permanently stops reminders.

The wording of the reminder is as follows:

This patient with Atrial Fibrillation is at raised risk of stroke. Anticoagulation is recommended unless contraindicated.

Please indicate your intended management:

1. I am not a clinician able to initiate anticoagulant therapy.

2. We do not have time to decide during this consultation over starting anticoagulation.

3. The patient is already taking anticoagulation, so no action needed, but I will apply the code ‘Anticoagulation monitoring’ to stop further alerts appearing.

4. We intend to start anticoagulation.

5. We have decided not to start anticoagulation because (tick all that apply):

* Evidence of atrial fibrillation (or history of it) is absent/inconclusive

* Patient preference (after benefits have been explained & risks discussed)

* Concern over on-going risk of serious haemorrhage

* Other reason/s (which I am recording in the notes)

Note - changes may be made to wording before the main phase, dependent on pilot results.

#### Intervention implementation

The intervention will be delivered into practices remotely over the EMIS Web system, and then activated on day 1 of the trial. Participant practices will receive information about the intervention from the study team prior to the study. This is a pragmatic trial, in which we will test the electronic intervention in routine practice. Intervention practices will agree to invite all patients on the list produced by the tool at the start of the trial, excluding those clearly unsuitable, for a consultation to discuss OAC therapy. The invitations will include an information sheet, provided by the study team, which gives patients details about AF and anticoagulation. Invitations will be sent within the first week of the trial, with reminders to non-attendees after one month.

Additionally, from day 1 of the trial screen reminders will appear in the medical records of eligible patients. Both the lists and screen reminders aim simply to promote current UK guidelines, and not novel management of AF [19].

### Control practices

Control practices will continue with usual care, which as of April 2012 included requirements placed on practices by the Quality and Outcomes Framework (QOF) [20]. The control practices will have the trial software installed, but this will run in a non-active format. There will be no eligible list generated and no screen reminders. The software will record the number of patients eligible for anticoagulation, the number receiving treatment and other secondary study outcomes throughout the trial.

### Study outcomes

#### Primary outcome

The primary outcome will be the proportion of patients with diagnosed AF, and eligible for anticoagulation under recent guidelines (NICE TA249) [19], who are prescribed therapy 6 months after the start of the intervention.

#### Secondary outcomes

Secondary outcomes include the following:

1. The proportion of patients with CHADS_2_ score ≥2 prescribed anticoagulants. CHADS_2_ is a commonly used scoring system for stroke risk in atrial fibrillation [8].

2. A record of inappropriate care or prescribing related to anticoagulation in patients with AF.

3. The incidence of stroke, transient ischaemic attack (TIA), or other major thromboembolism during the trial, in patients with AF eligible for OAC.

4. The incidence of major haemorrhage during the trial in patients with AF eligible for OAC.

The denominator for the primary outcome will be all of those eligible for OAC therapy at the end of the 6-month trial period. This will include those whose eligibility has arisen since the start of the trial, for instance through reaching the 65- or 75-year age thresholds, or through a new diagnosis of a condition relevant to eligibility. It is important that the size of the denominator population is not influenced by use of the tool, as this could lead to ascertainment bias. We will therefore exclude from the outcome denominator those whose eligibility has arisen during the study purely as a result of improved recognition of diabetes, hypertension, heart failure, or thromboembolic stroke by the AURAS-AF tool, as the control arm will not be influenced by this improved recognition (see ‘Intervention’ section above). This group will be identifiable through the recording of responses to screen prompts. Patients diagnosed with these conditions independently of the tool will not be excluded. Similarly, cases where an ‘Atrial Fibrillation Resolved’ code is applied (effectively removing the patient from the study population) will be retained in the denominator if this decision resulted directly from the use of the AURAS-AF tool in the intervention arm.

### Allocation

The 46 EMIS Web practices that consent to participate will be randomised into two equal groups (intervention and control). We will use balanced allocation due to the small number of practices. An independent statistician will randomise practices using block randomisation, stratifying for practice list size and the proportion of their eligible population prescribed OACs (groups separated by the median).

### Data collection

The primary data collection will use the capabilities of EMIS Web. The research team will enter into data sharing agreements with practices allowing us to securely access anonymous outcome data over an N3 NHS (secure) internet connection. Proportions of patients prescribed OACs, and incidence of stroke, TIA, other major thromboembolism and major haemorrhage in the TA249 eligible sample will be collected in this way. We will collect these outcomes at baseline, 3 months into the trial (for the data monitoring committee), at 6 months (at the end of the intervention phase) and after 12 months (see Additional file 1).

We shall additionally ask practices - both intervention and control - to fill in *pro forma* providing further information on strokes, TIAs, other major thromboembolisms and major haemorrhages. The number of these required from each individual practice is expected to be small. These will include information about the anticoagulation status (including international normalised ratio if available) at the time of the event and duration on therapy. The *pro forma* data will help to attribute outcome events to decisions around prescribing and the possible influence of the AURAS-AF software on these decisions. Intervention practices will also be asked to complete *pro forma* providing details of patients’ contact with the practice during the study that is relevant to anticoagulation. This will include responses to the initial invitation to consultation, and reasons behind decisions over therapy where discussions occurred. The main outcome data will be extracted immediately following the intervention phase, including the *pro forma*, with a second extract after a further 6 months. Finally, we ask practices to report any inappropriate care or prescribing in the AF population to the study team within one week of its occurrence.

#### Statistical analyses

We will compare baseline characteristics of randomised practices and their demographic characteristics between trial arms. For all randomised practices, we will report the aggregated number of eligible people identified at the start of the study, the proportion of these invited (with reasons if not invited), the proportion of attendees, and the proportion of those attending that commence anticoagulation by the end of the trial.

The anonymous data on anticoagulant prescribing from each practice will be aggregated and analysed using analysis of covariance (ANCOVA). We shall compare proportions of eligible patients who are using anticoagulation after 6 months, between intervention and control arms (primary outcome), adjusting for baseline values. We shall also compare this measure using data from one year after the start of the study. We will assess whether any practice characteristics have an impact on the intervention effect using multivariable regression. Incidence rates of stroke, TIA, other major thromboembolisms and major haemorrhage (secondary outcomes) will be compared between arms [23].

### Pilot study

A 3-month pilot study will be conducted before the main phase of the trial. The pilot is designed to assess limitations in the usability and appropriateness of the intervention and trial material. Main outcomes will be improvements to the electronic tool and trial material before the main phase of the study.

We will install the intervention tool into three pilot practices. These practices will take on the study procedures: briefly, having the list of those eligible produced, inviting patients to consultation, reminding non-attendees after one month, and having the screen reminders activated. We shall interview two patients and two staff (one general practitioner (GP) and one other member) per practice. These interviews will focus on the usability and appropriateness of the electronic tool and patient material. Finally, we will remotely extract outcome data approximately one month into the pilot to test the extraction system.

### Process evaluation

The process evaluation, running in parallel with the intervention phase, will produce important study outcomes. The process evaluation aims:

1. To examine patient and practitioner views of electronic reminder systems in primary care, and their impact on consultations and practice.

2. To investigate barriers and enablers to the successful take up of an electronic audit and reminder tool by primary care teams.

3. To explore any barriers to recommended prescribing in AF that persist after the use of an electronic audit and reminder system.

We will collate data on the responses made to the screen reminders throughout the intervention period (that is, option chosen in response to a screen message). This will be logged by the intervention tool, and accessed remotely after the intervention phase. These data will allow us to study the suitability of the reminders.

The core of the process evaluation will be qualitative work with GPs and patients in intervention practices. We shall carry out semi-structured interviews with 10 GPs and up to 30 patients (a maximum of 1 and 5 respectively per practice), stopping if data saturation is reached (see Additional files 2 and 3). Intervention practices will identify patients and GPs eligible for interview, namely those who have been in contact with the intervention. We seek GP interview participants with maximum variability across practice list size and the location of the practice.

Interview participants will give individual consent, in addition to practice consent. GP interviews will focus on their views about the software tool, concentrating on impacts of the screen reminders on the consultation process, how well the software promotes best practice prescribing and any persisting perceived barriers to prescribing. We shall interview patients who have had a consultation to discuss anticoagulation as a result of the trial, stratifying by whether attendance resulted from the invite letter or an opportunistic reminder. We seek patients views on screen reminders in primary care, for example their thoughts on the change of subject of the consultation that they create [16], and on opportunistic health promotion - compared to the systematic approach of the letters. Interview transcripts will be analysed thematically and entered into a specialist software package (NVivo 9). We will organise and analyse emergent themes using the method of constant comparison.

### Sample size calculation

We have powered the study to detect changes in one of the secondary outcomes - change in proportion with CHADS_2_ score ≥2 who are prescribed anticoagulants. This is a smaller group than the denominator for the primary outcome, hence the need for greater power, but is important given its prominence in the QOF. We estimate a sample of 46 practices will be needed for the trial, accounting for clustering, variation in cluster size and 10 percent withdrawals [24]. This will give 95 percent power to detect a relative difference of 25 percent in proportion of eligible people who are treated between the trial arms, at 5 percent significance level.

The cluster size (*m* - number of patients with CHADS_2_ ≥2 in each practice) is estimated at 54 (based on mean list size and AF prevalence in the QOF [25], and recent CHADS_2_ estimates [8]). There is no relevant estimate of minimum change due to intervention; therefore we assumed 25 percent relative increase based on preliminary discussions. We estimated baseline prescribing of 53 percent [8], therefore 66 percent at follow-up. Assuming an intra-class correlation coefficient (ICC) of 0.03 (using a published ICC for inappropriate prescribing in the elderly [26]) and coefficient of variation (*cv*) of cluster size of 0.6 (based on a sample of THIN data on file; mean AF patients per practice = 119, standard deviation = 72) we estimate the design effect to be 3.17 (1 + ((1 + *cv*[2]) *m* - 1) x ICC) [27]. The initial total sample size, ignoring clustering is 720 (total in both arms) [28], therefore 2,282 accounting for the design effect. Including a 10 percent withdrawal this equates to 46 practices.

### Ethical considerations

As a cluster randomised trial, with practices as participants, the trial has specific ethical considerations. We seek informed consent to participate from the practices, with a practice ‘guardian’ consenting to trial entry [21,22] on the basis that the guardian will act for the good of the cluster; benefits and harms of entry to the trial will not exceed non-entry and the intervention will not be too onerous or contentious for cluster members [22]. Interview participants will give individual informed consent. We shall use data from the primary care medical records; however we will only receive anonymised *pro forma* data or data aggregated to a practice level. Finally, we shall employ a data monitoring committee to monitor study progress for safety, notably the secondary outcomes (inappropriate prescribing, stoke, TIA, and haemorrhages).

The study protocol and associated material have received a full favourable opinion from the NRES Committee South Central - Berkshire (REC number 13/SC/0026); and national NHS research and development approval. We have registered the trial on the International Standard Randomised Controlled Trial Number Register (ISRCTN55722437) and the UK Clinical Research Network portfolio. We have developed the full study protocol in line with SPIRIT guidelines (full protocol available on request) [29], and will conduct and report the trial in line with the CONSORT extension to cluster randomised trials [24].

## Discussion

AURAS-AF investigates whether a reminder intervention, using electronic primary care records, can safely increase anticoagulant uptake in a high stroke risk group with AF. This has been recognised as an important area for stroke prevention, and one that has lagged behind improvements in other areas of stroke care [8,30]. A number of barriers to anticoagulation have been proposed, however, their precise importance remains unclear [12]. Given the relative complexity of stoke risk scoring tools, and recently changing guidelines for anticoagulant eligibility, clinician uncertainty may play a role [14]. The use of automated tools that harness electronic data in primary care may clarify which patients with AF are eligible for OAC and flag this risk during routine care.

Electronic reminders are now commonplace in UK primary care, but the evidence to support their use is disproportionately low. Few have been assessed in randomised trials [31,32], and questions remain as to whether the quality of the underlying data is sufficient to risk assess patients for serious outcomes [8,33]. The evidence behind consultation based reminders is especially important when considering their potential for harm, or more specifically their disruptiveness and/or irrelevance to the clinical problem at hand. It has been proposed that such reminders may reduce patient autonomy; lengthen consultations excessively; and lead to clinician reliance [16-18]. Finally, for AF, the prompts could rush clinicians into unsafe prescribing. There is little substantive evidence to support, or dispel, these concerns. Our process evaluation of the AURAS-AF intervention will provide valuable data, especially qualitative, on the safety and suitability of electronic reminders in this area of care. We anticipate that prescribing may in fact be safer through use of the tool - with the most recent national eligibility criteria applied systematically to patient data - but this assumption requires confirmation though this research [19]. Reports of inappropriate prescribing in the study will therefore be important outcomes.

Finally, the AURAS-AF trial will test a relatively new approach to conducting primary care research. Recent studies have utilised primary care databases to facilitate randomised controlled trials [32,34]. We will use the capabilities of a web-based clinical system both to deploy a primary care intervention and to collect outcome data. There has been a very recent move to generate data for cross-sectional analyses through the anonymous extraction from medical records via this method [35,36]. Use of this extraction process for a randomised trial may substantially reduce costs of data collection and provides a novel platform for the study of interventions and processes of care in a range of clinical areas.

## Trial status

The intervention tool has been developed and the pilot study began on 21 June 2013. Recruitment for the main phase began on 1 July 2013, with an anticipated start date of 1 December 2013.

## Abbreviations

AF: Atrial fibrillation; EMIS: Egton Medical Information Systems; GP: General practitioner; ICC: Intra-class correlation coefficient; NICE: National Institute for Health and Clinical Excellence; NIHR: National Institute for Health Research; NRES: National Research Ethics Service; NYHA: New York Heart Association; OAC: Oral anticoagulant; PCRN: Primary Care Research Network; QOF: Quality and Outcomes Framework; TIA: Transient ischaemic attack

## Competing interests

Authors declare that they have no competing interests.

## Authors’ contributions

TH, DF, TM, MF, NQ, RH, DL and KK conceived the study design and obtained funding; AD, TH, DF, TM, MF, NQ, RH, DL, KK and JJ contributed to preparation for the study, developed the quantitative study design and obtained ethical approval; JH, AD and TH developed the qualitative study design; all authors contributed to the final version of the protocol. All authors read and approved the final manuscript.

## Supplementary Material

Additional file 1: Read code lists. Click here for file

Additional file 2: Practitioner topic guide for qualitative interviews. Click here for file

Additional file 3: Patient topic guide for qualitative interviews. Click here for file

## References

[B1] Office for National Statistics Deaths registered in England and Wales in 2010, by cause, In Book Deaths registered in England and Wales in 2010, by cause 2011 London, England: Office for National Statistic

[B2] Adamson J Beswick A Ebrahim S Is stroke the most common cause of disability? J Stroke Cerebrovasc Dis 2004 13 171 177 10.1016/j.jstrokecerebrovasdis.2004.06.003 17903971

[B3] Rothwell PM Coull AJ Giles MF Howard SC Silver LE Bull LM Gutnikov SA Edwards P Mant D Sackley CM Farmer A Sandercock PA Dennis MS Warlow CP Bamford JM Anslow P Oxford Vascular Study: change in stroke incidence, mortality, case-fatality, severity, and risk factors in Oxfordshire, UK from 1981 to 2004 (Oxford Vascular Study) Lancet 2004 363 1925 1933 10.1016/S0140-6736(04)16405-2 15194251

[B4] Wolf PA Abbott RD Kannel WB Atrial-fibrillation as an independent risk factor for stroke - the framingham-study Stroke 1991 22 983 988 10.1161/01.STR.22.8.983 1866765

[B5] Lin HJ Wolf PA Kelly-Hayes M Beiser AS Kase CS Benjamin EJ D’Agostino RB Stroke severity in atrial fibrillation. The Framingham Study Stroke 1996 27 1760 1764 10.1161/01.STR.27.10.1760 8841325

[B6] Hart RG Halperin JL Atrial fibrillation and stroke: concepts and controversies Stroke 2001 32 803 808 10.1161/01.STR.32.3.803 11239205

[B7] Lip GY Edwards SJ Stroke prevention with aspirin, warfarin and ximelagatran in patients with non-valvular atrial fibrillation: a systematic review and meta-analysis Thromb Res 2006 118 321 333 10.1016/j.thromres.2005.08.007 16198396

[B8] Holt TA Hunter TD Gunnarsson C Khan N Cload P Lip GYH Risk of stroke and oral anticoagulant use in atrial fibrillation: a cross-sectional survey Brit J Gen Pract 2012 62 e710 e717 10.3399/bjgp12X656856 23265231 PMC3459779

[B9] Cowan C Healicon R Robson I Long WR Barrett J Fay M Tyndall K Gale CP The use of anticoagulants in the management of atrial fibrillation among general practices in England Heart 2013 99 1166 1172 10.1136/heartjnl-2012-303472 23393083 PMC3717828

[B10] Ogilvie IM Newton N Welner SA Cowell W Lip GY Underuse of oral anticoagulants in atrial fibrillation: a systematic review Am J Med 2010 123 638 645 e634 10.1016/j.amjmed.2009.11.025 20609686

[B11] Nieuwlaat R Capucci A Lip GY Olsson SB Prins MH Nieman FH Lopez-Sendon J Vardas PE Aliot E Santini M Crijns HJ Antithrombotic treatment in real-life atrial fibrillation patients: a report from the Euro Heart Survey on Atrial Fibrillation Eur Heart J 2006 27 3018 3026 10.1093/eurheartj/ehl015 16731536

[B12] Riva N Smith DE Lip GY Lane DA Advancing age and bleeding risk are the strongest barriers to anticoagulant prescription in atrial fibrillation Age Ageing 2011 40 653 655 10.1093/ageing/afr128 21951858

[B13] Olesen JB Lip GYH Lindhardsen J Lane DA Ahlehoff O Hansen ML Raunso J Tolstrup JS Hansen PR Gislason GH Torp-Pedersen C Risks of thromboembolism and bleeding with thromboprophylaxis in patients with atrial fibrillation: a net clinical benefit analysis using a ‘real world’ nationwide cohort study Thrombosis and haemostasis 2011 106 739 749 10.1160/TH11-05-0364 21789337

[B14] Stott D Dewar R Garratt C Griffith K Harding N James M Lane D Petty D Smith P Somerville M RCPE UK Consensus Conference on ‘approaching the comprehensive management of atrial fibrillation: evolution or revolution?’ J R Coll Physicians Edinb 2012 42 suppl 18 3 4 22518389 10.4997/JRCPE.2012.S01

[B15] Holt TA Thorogood M Griffiths F Changing Clinical Practice Through Patient Specific Reminders Available at the Time of the Clinical Encounter: systematic Review and Meta-Analysis J Gen Intern Med 2012 27 974 984 10.1007/s11606-012-2025-5 22407585 PMC3403145

[B16] Getz L Sigurdsson JA Hetlevik I Is opportunistic disease prevention in the consultation ethically justifiable? BMJ 2003 327 498 10.1136/bmj.327.7413.498 12946974 PMC188390

[B17] Mitchell E Sullivan F A descriptive feast but an evaluative famine: systematic review of published articles on primary care computing during 1980–97 BMJ 2001 322 279 282 10.1136/bmj.322.7281.279 11157532 PMC26582

[B18] Chambers CV Balaban DJ Carlson BL Grasberger DM The effect of microcomputer-generated reminders on influenza vaccination rates in a university-based family practice center J Am Board Fam Pract 1991 4 19 26 1996510

[B19] National Institute for Health and Care Excellence (NICE) Dabigatran etexilate for the prevention of stroke and systemic embolism in atrial fibrillation (TA249) 2012 London, England: NICE

[B20] Smith SA Gorman CA Murphy ME Zimmerman BR Huschka TR Rizza RA Dinneen SF Naessens JM Impact of a diabetes electronic management system on the care of patients seen in a subspeciality diabetes clinic Diabetes Care 1998 21 972 976 10.2337/diacare.21.6.972 9614616

[B21] Weijer C Grimshaw JM Taljaard M Binik A Boruch R Brehaut JC Donner A Eccles MP Gallo A McRae AD Ethical issues posed by cluster randomized trials in health research Trials 2011 12 100 10.1186/1745-6215-12-100 21507237 PMC3107798

[B22] Edwards SJ Braunholtz DA Lilford RJ Stevens AJ Ethical issues in the design and conduct of cluster randomised controlled trials BMJ 1999 318 1407 1409 10.1136/bmj.318.7195.1407 10334756 PMC1115783

[B23] Ng HK Tang ML Testing the equality of two Poisson means using the rate ratio Stat Med 2005 24 955 965 10.1002/sim.1949 15532090

[B24] Health and Social Care Inforamtion Centre The Quality and Outcomes Framework 2010/11 http://www.qof.hscic.gov.uk/

[B25] Campbell MK Piaggio G Elbourne DR Altman DG Consort 2010 statement: extension to cluster randomised trials BMJ 2012 345 e5661 10.1136/bmj.e5661 22951546

[B26] Cosby RH Howard M Kaczorowski J Willan AR Sellors JW Randomizing patients by family practice: sample size estimation, intracluster correlation and data analysis Fam Pract 2003 20 77 82 10.1093/fampra/20.1.77 12509376

[B27] Eldridge SM Ashby D Kerry S Sample size for cluster randomized trials: effect of coefficient of variation of cluster size and analysis method Int J Epidemiol 2006 35 1292 1300 10.1093/ije/dyl129 16943232

[B28] Machin D Campbell MJ Statistical tables for the design of clinical trials 1987 England: Blackwell Scientific Oxford

[B29] Chan AW Tetzlaff JM Gotzsche PC Altman DG Mann H Berlin JA Dickersin K Hrobjartsson A Schulz KF Parulekar WR Krleza-Jeric K Laupacis A Moher D SPIRIT 2013 explanation and elaboration: guidance for protocols of clinical trials BMJ 2013 346 e7586 10.1136/bmj.e7586 23303884 PMC3541470

[B30] Lee S Shafe AC Cowie MR UK stroke incidence, mortality and cardiovascular risk management 1999–2008: time-trend analysis from the General Practice Research Database BMJ Open 2011 1 e000269 22021893 10.1136/bmjopen-2011-000269 PMC3211058

[B31] Holt TA Thorogood M Griffiths F Munday S Friede T Stables D Automated electronic reminders to facilitate primary cardiovascular disease prevention: randomised controlled trial Br J Gen Pract 2010 60 e137 e143 10.3399/bjgp10X483904 20353659 PMC2845504

[B32] Dregan A Van Staa T Mcdermott L McCann G Ashworth M Charlton J Wolfe C Rudd A Yardley L Gulliford M Cluster randomized trial in the general practice research database: 2. Secondary prevention after first stroke (eCRT study): study protocol for a randomized controlled trial Trials 2012 13 181 10.1186/1745-6215-13-181 23034059 PMC3570277

[B33] Ashworth M Medina J Morgan M Effect of social deprivation on blood pressure monitoring and control in England: a survey of data from the quality and outcomes framework BMJ 2008 337 a2030 10.1136/bmj.a2030 18957697 PMC2590907

[B34] Dregan A Toschke MA Wolfe CD Rudd A Ashworth M Gulliford MC Utility of electronic patient records in primary care for stroke secondary prevention trials BMC Public Health 2011 11 86 10.1186/1471-2458-11-86 21299872 PMC3041663

[B35] Mathur R Noble D Smith D Greenhalgh T Robson J Quantifying the risk of type 2 diabetes in East London using the QDScore: a cross-sectional analysis Brit J Gen Pract 2012 62 522 523 10.3399/bjgp12X652553 PMC3459773 23265225

[B36] Badrick E Hull S Mathur R Shajahan S Boomla K Bremner S Robson J Health equity audits in general practice: a strategy to reduce health inequalities Prim Health Care Res Dev 2013 4 1 16 10.1017/S1463423612000606 23375244

