# Supplementary material for: AUtomated Risk Assessment for Stroke in Atrial Fibrillation (AURAS-AF) - an automated software system to promote anticoagulation and reduce stroke risk: study protocol for a cluster randomised controlled trial
Source: Trials. 2013 Nov 13;14:385. doi: 10.1186/1745-6215-14-385 (PMC4225760; doi:10.1186/1745-6215-14-385)
Supplement: Additional file 1: — Read code lists. [file 1745-6215-14-385-S1.docx]

| previous | Thromboembolic stroke | G63y0%; G63y1%; G64%; G65% (excluding G655); G6760; G6W; G6X; G665; G666; Gyu63; Gyu64; Gyu65; Gyu66; Gyu6G; ZV12D |
| --- | --- | --- |
|  | Unrecorded thromboembolic stroke | G66%, ZV125 |
|  | Transient ischaemic attack | G65..- G654.; G656.- G65zz; ZV12D; Fyu55 |
|  | Systemic embolism | G74% |
|  | Excluded if haemorrhagic stroke | G61%; Gyu62; Gyu6F |
|  | Excluded if major haemorrhage | G60..; 1C6..; TA0..; S62..; S70..; 851..; L36..; G62..; G61..; L3A..; D31..; J68..; S63..; L11..; 158..; L10..; 1582; 98BY.; G615.; J681.; C063.; L361.; S631.; 14AF.; 196C.; 1583; S62z.; G61z.; S707.; L091.; S628.; S621.; J68z.; 98BN.; 98BR.; S703.; G602.; R048.; 158Z.; L10y.; G612.; 1581; K167.; G623.; G616.; G603.; G620.; 98Ca.; S629.; S70z.; 98BO.; 1584; G601.; S625.; S62A.; G699.; S63z.; 1581; L114.; G604.; K221.; L360.; L10z.; S623.; J680.; G611.; L11z.; J573.; G682.; 98BM.; 14CA.; G60z.; L36z.; S708.; 98CZ.; 98CY.; L111.; G605.; 2DE7.; G60X.; G614.; F4K7.; 98BX.; 98BW.; S627.; G612.; G610.; S620.; S630.; 98BP.; G611.; G600.; G621.; G8y0.; G62z.; 14CD.; 196B.; 14c1.; SP21.; L362.; L115.; 1C6Z.; L11y.; G622.; 98BQ.; G680.; G617.; S622.; F42y.; S704.; G61X.; S702.; 1582; G681.; SK02.; S705.; G613.; L081.; G606.; 15A1.; 15A6.; L113.; S626.; G618.; S624.; S714.; G77z0; L10zz; R0630; J14y3; L0701; Lyu4D; S6306; S7140; K59yy; L1150; S6250; L111z; L091z; S6221; K2752; L0421; K2861; J11y2; J1203; S6236; S6302; J1500; S6300; S6240; J1213; S6246; S6233; S6215; 7M0U4; L362z; J1411; S6200; S625z; S6254; S6291; S6290; L0441; F42y4; L113z; J14y1; J13y1; G61X0; S6245; F4G32; S6313; L1132; L0501; J1201; L3600; S6304; S6312; L11z1; L1112; S62A1; F42y3; L0431; FyuH4; S6234; S6315; S62A0; S6314; C0630; K59yx; S630z; F4Ey0; Lyu47; F4C71; SP211; S622z; S6310; S6205; K16y2; L361z; SP210; L11z0; S7141; S7511; S6216; F42y1; Gyu6B; S6220; SP214; S6203; F4C72; L3601; Gyu62; S6211; S620z; L1110; J1103; S6316; L115z; L10y2; S624z; S6311; L3612; S6242; L11z2; S6303; L11y1; L0521; L3602; S6204; S6243; L3611; L3610; J573z; J68zz; J11y3; L11y2; J1413; G61X1; S6241; Ryu07; J1113; 7F227; L0711; L1141; F42y5; L1151; S6202; S7601; L1130; L0601; L0511; L10z2; L10z1; L0401; S6253; S6232; S6251; L0721; S623z; L0611; R0631; S6225; L0411; Lyu3E; L360z; Gyu61; K55y3; C1542; J68z0; L1131; L11y0; J1301; J14yy; J68z2; J11y1; S6235; S621z; J5731; J5730; K2211; S6226; L3621; J1403; L0621; J13yy; J13y3; F4H41; J12yy; J1311; L1140; J11yy; J1211; Lyu20; F4045; K5311; J1111; K1381; L1111; J12y3; L10yz; K286w; J1101; S7611; K2864; L11yz; SP213; S6224; S6301; L114z; Gyu6F; J1303; S6212; L10z0; S6252; S6305; S6223; L3622; L10y0; H5y00; S6244; S6214; F4K28; S6222; K221z; L1152; S631z; S6256; J68z1; S6255; L1142; S6231; L10y1; Ryu02; S6201; L3620; J1401; C12y1; 70170; Gyu60; S6230; S6213; K56y1; J1313; S6206; J12y1; L11zz; S6210; TA0.. |
| left ventricular ejection fraction below 40% | | G58..%; G1yz1; 662f. – 662i.; 585f.; G5yy9 |
| symptomatic heart failure of New York Heart Association (NYHA) class 2 or above | |  |
| Heart failure (undiagnosed) | | Echocardiography - poor LV function; 33BA Impaired left ventricular function; 33BB Left ventricular ejection fraction (Value <40%); Echocardiogram ejection fraction (Value <40%); Heart failure lifestyle plan commenced; Heart failure monitoring - co-medications; Heart failure monitoring - co-morbidities; Heart failure monitoring - multiple readmissions; Heart failure monitoring - palliative care; Heart failure monitoring - psychological issues; Heart failure monitoring - social issues; Heart failure monitoring - specialist clinical needs; Heart failure monitoring - unstable symptoms |
| age 75 years or older | | n/a |
| age 65 years or older with one of: | diabetes mellitus | C10..; C109J; C109K; C10C.; C10D.; C10E.%; C10F.% (Excluding C10F8); C10G.%; C10H.%; C10M.%; C10N.%; PKyP. |
|  | diabetes mellitus (undiagnosed) | Not on Diabetic Register; Do not have Gestational Diabetes in past 9 months; Do not have Diabetes Excluded or Normal Glucose Tolerance Test; Do not have Impaired Glucose Tolerance coded  But are either  on Diabetic Medication (BNF Section 6.1.1 or 6.1.2)  OR  Group A: Those whose most recent level was either >=7.0mmol/L fasting OR >=11.1mmol/L random where a later HbA1c does not exist or if it does it is >=48  OR  Group B: Those whose most recent level was >=7.0mmol/L irrespective of fasting; random; or non-specific code used where a later HbA1c does not exist or if it does it is >=48  OR  Group C; those with an Hba1c >=48 mmol/mol (however long ago) with no subsequent Hba1c value lower than this and no subsequent blood glucose level (any code) less than 7.0mmol/L. |
|  | coronary artery disease | G3... – G309.; G30B. - G330z (excluding G310.); G33z. - G3401; G342. – G35X.; G38.. – G3z..; Gyu3.% (excluding Gyu31) |
|  | hypertension | G2...; G20..%; G24.. - G2z.. (Excluding G24z1; G2400; G2410; G27..); Gyu2.; Gyu20 |
|  | hypertension (undiagnosed) | Not on Hypertension Register and ACE Inhibitors and Angiotensin receptor blockers (BNF section 2.5.5.1 and 2.5.5.2) and NO Heart Failure Codes QOF)  Or  Calcium channel blockers (BNF section 2.6.2) and NO IHD Codes (QOF)  Or  Thiazide diuretics (BNF Section 2.2.1);  Or  Alpha blockers (BNF Section 2.5.4)  OR  Beta blockers (BNF Section 2.4) |
