# Supplementary material for: AUtomated Risk Assessment for Stroke in Atrial Fibrillation (AURAS-AF) - an automated software system to promote anticoagulation and reduce stroke risk: study protocol for a cluster randomised controlled trial
Source: Trials. 2013 Nov 13;14:385. doi: 10.1186/1745-6215-14-385 (PMC4225760; doi:10.1186/1745-6215-14-385)
Supplement: Additional file 2: — Practitioner topic guide for qualitative interviews. [file 1745-6215-14-385-S2.docx]

**Practitioner Topic Guide**

**A qualitative study of general practitioner and practice staff views on a new intervention system in primary care to reduce stroke risk in atrial fibrillation**

1. **Background: The doctor, the practice and AF**

Could you tell me a little about yourself and about your work in this practice *(e.g. when qualified, time working in this practice, population etc.).*

How often do you come across people with AF during your working week?

Do you have any specific training in treating patients with AF?

1. **Attitudes to use of anticoagulant therapy in treating patients with AF**

*There is widespread underuse of anticoagulants to treat people with AF in Europe. In the UK, just over 50% of people with AF and at risk of stroke are taking anticoagulants.*

How important do you think anticoagulant therapy is in treating patients with AF?

In what ways do you try and encourage your patients to take anticoagulants?

Which anticoagulants do you prescribe?

What do you consider to be the main barriers to uptake?

Can you give me some examples from your practice which illustrate patient attitudes to taking anticoagulants *(positive and negative)*

1. **The intervention**

*As you are aware, we have been trialling the use of an electronic reminder system to identify patients who have AF but who up until now have not been prescribed anticoagulant medication. The goal is to increase uptake of medication by discussing with them the pros and cons of anticoagulant therapy.*

What are your thoughts on taking part in the trial?

*The trial intervention comprises three components. I’d now like to ask you to comment on each of these:*

1. Identifying patients

*A search module generates a list of patients eligible to take part in the trial. GPs are asked to send letters inviting these patients to a consultation to discuss the pros and cons of anticoagulation therapy.*

How successful has the module been in identifying eligible patients? /Do you feel that the systematic invitation of patients is an efficient way to use practice resources?

Do you feel that the quality of primary care data is sufficiently high to facilitate the pre-identification of patients eligible for treatment? Do you feel any patients will miss out necessary care if pre-stratification were adopted?

What inclusion/exclusion criteria have you used in deciding which patients to invite to take part? Why?

How did you feel about completing the final table giving reasons for treatment decisions for people identified at baseline?

1. Screen messages

*A screen message reminding clinicians of the need for anticoagulation unless contraindicated appears whenever the electronic record of a patient currently on the list is opened.*

Were there any problems in setting up this system in your practice?
The screen offers GPs a number of options in relation to intended management of identified patients. What changes, if any, would you make to these options?
How has the system worked in practice? Give details/examples.

How time consuming was dealing with the pop-ups?

How did the pop-ups affect the flow of consultations with patients about other medical issues? Give details/examples

Has the presence of the on-screen reminders impacted on the time given to discuss anticoagulation and the patients autonomy to make treatment decisions? Do you feel that this has resulted in any inappropriate prescribing events?

Have you any knowledge of the reminders being misdirected – at staff unable to make prescribing decisions, affecting their workload

*You were sent an information sheet summarising the current evidence on benefits versus risks of anticoagulation in high risk groups.*

In what ways did this information inform your consultation with patients?

1. **Patient response***Invited patients*

How receptive have patients been in accepting the invitation to discuss anticoagulant therapy?

Tell me how you discussed the issue of stroke risk and anticoagulant therapy in the consultation.

Can you give me an example of a consultation where a patient responded positively to going on anticoagulants?

Can you give me an example of a consultation where a patient rejected the use of anticoagulants? What reasons did he/she give?

How successful has the intervention been in changing attitudes to anticoagulant use?

*Patients identified through electronic reminder in consultation*

How receptive have patients been to discussing the use of anticoagulation therapy as part of a consultation for a non-AF related condition?

Can you give me an example of a consultation in which this happened?
What was the outcome?

1. **Thoughts on intervention and care of people with AF in primary care**

How effective do you feel the use of an electronic monitoring system such as this is in encouraging greater uptake of anticoagulation therapy?

How feasible would it be to implement this system in all practices? What obstacles would you envisage?

What else can we do in primary care to increase patient awareness of stroke risk and the importance of taking anticoagulant medication?

What should ‘good care’ for people with AF look like?

Will AF become a bigger concern in the future (how/why)? How might this impact on your practice?

On balance, which approach do you think is more appropriate in discussing anticoagulation with patients in primary care: systematic invitation or the opportunistic identification (using reminders? Are both approaches needed? Consider how it fits into a routine care environment / most effectively uses practice resources

1. **Final questions/comments**

We’ve reached the end of our interview. Is there anything else you’d like to add that we might have missed out?
