# Supplementary material for: AUtomated Risk Assessment for Stroke in Atrial Fibrillation (AURAS-AF) - an automated software system to promote anticoagulation and reduce stroke risk: study protocol for a cluster randomised controlled trial
Source: Trials. 2013 Nov 13;14:385. doi: 10.1186/1745-6215-14-385 (PMC4225760; doi:10.1186/1745-6215-14-385)
Supplement: Additional file 3: — Patient topic guide for qualitative interviews. [file 1745-6215-14-385-S3.docx]

**Patient Topic Guide**

**A qualitative study of patient views on a new intervention system in primary care to reduce stroke risk in atrial fibrillation**

1. **Background**

How long have you had AF?

How did you feel when you were first diagnosed? What were your main concerns? *(probe: risk of stroke)*
What information were you given about AF?

What treatment options did your doctor explore with you? Did your doctor explain the pros and cons of different treatments and outline any risks?
What decisions did you make about treatment?
Were there any medications offered which you decided not to take? Why? *(probe: attitudes towards anticoagulants and perceived barriers to taking medication)*

How has your medication changed over time? What do you take now for your AF?

Tell me about any side-effects you have experienced.

How often do you see your GP about your AF?

1. **Intervention**

*You recently spoke to your doctor about the pros and cons of taking anticoagulant (blood thinning) medication to minimise your risk of stroke.*

How did this meeting come about? Did you receive a letter from your GP inviting you to come and see him about this? If so, how useful was the patient information sheet you received about anticoagulation therapy?

How did you feel receiving the letter? *(probe: concerns; feeling reassured that GP caring)*

Or did your GP bring up the topic when you came to see him for other reasons? Can you tell me about the consultation with your GP - What did you talk about?

What decisions did you make about taking anticoagulants following the meeting? How long did it take you to decide? Who else did you talk to about your decision?

Did the invitation from your GP (or the presence of a pop-up reminder on your doctor’s computer), influence your decision? How? When you were with your doctor, did you notice a reminder pop up on his/her computer during the consultation? What did your doctor do? Was he able to deal with it at the time? Where the practitioner was unable to act on it- i.e. unable to prescribe / proceed with discussion?

*(If now on anticoagulants)*

You’ve decided to go on anticoagulants since seeing your doctor. In what ways did the consultation with your GP help you reach this decision? What type of anticoagulants are you taking?
Tell me what it’s like being on them *(probe: side-effects, regular blood tests, increased risk of bleeding, impact on life etc.)*

What do you see as the main advantage/s of taking anticoagulants? Any disadvantages?

*(If decided against anticoagulants)*

You’ve decided not to take anticoagulants since seeing your doctor. Can you tell me why?

1. **General thoughts on intervention**

*We have been trialling the use of an electronic reminder system to identify patients such as yourself who have AF but who (up until now) have not been taking anticoagulant medication. The idea is to encourage more people to go on anticoagulants to help prevent the risk of stroke by providing an opportunity for GPs to discuss the pros and cons of medication with their patients.* *(If necessary, briefly describe how intervention system works).*

What do you think of this intervention?

(If pop-up reminder initiated discussion of anticoagulants) How did you feel when your GP started to talk to you about anticoagulation when you had come about a different issue? Could we ask something specifically about what the patient thinks of having the course of a consultation taken out of their hands (if they did in fact have a pop-up)??

What do you think are the main barriers to people with AF taking anticoagulants?

What else can we do to increase patients’ awareness of stroke risk and the importance of taking anticoagulant medication?

What should ‘good care’ for people with AF look like?

1. **The future**

What impact do you think AF might have on your life in future? Any concerns?

1. **Final questions/comments**

We’ve reached the end of our interview. Is there anything else you’d like to add that we might have missed out?
